# Retinofugal Projections Into Visual Brain Structures in the Bat *Artibeus planirostris*: A CTb Study

**DOI:** 10.3389/fnana.2018.00066

**Published:** 2018-08-08

**Authors:** Melquisedec A. D. Santana, Helder H. A. Medeiros, Mariana D. Leite, Marília A. S. Barros, Paulo Leonardo Araújo de Góis Morais, Joacil Germano Soares, Fernando V. L. Ladd, Jeferson S. Cavalcante, Judney C. Cavalcante, Miriam S. M. O. Costa, Expedito Silva Nascimento Jr.

**Affiliations:** ^1^Laboratory of Neuroanatomy, Department of Morphology, Federal University of Rio Grande do Norte, Natal, Brazil; ^2^Department of Zoology, Federal University of Pernambuco, Recife, Brazil; ^3^Laboratory of Neurochemical Studies, Department of Physiology, Bioscience Center, Federal University of Rio Grande do Norte, Natal, Brazil

**Keywords:** visual system, chiropteran, phyllostomidae, cholera toxin subunit b, retinal projections, pretectal region, superior colliculus, lateral geniculate nucleus

## Abstract

A well-developed visual system can provide significant sensory information to guide motor behavior, especially in fruit-eating bats, which usually use echolocation to navigate at high speed through cluttered environments during foraging. Relatively few studies have been performed to elucidate the organization of the visual system in bats. The present work provides an extensive morphological description of the retinal projections in the subcortical visual nuclei in the flat-faced fruit-eating bat (*Artibeus planirostris*) using anterograde transport of the eye-injected cholera toxin B subunit (CTb), followed by morphometrical and stereological analyses. Regarding the cytoarchitecture, the dorsal lateral geniculate nucleus (dLGN) was homogeneous, with no evident lamination. However, the retinal projection contained two layers that had significantly different marking intensities and a massive contralateral input. The superior colliculus (SC) was identified as a laminar structure composed of seven layers, and the retinal input was only observed on the contralateral side, targeting two most superficial layers. The medial pretectal nucleus (MPT), olivary pretectal nucleus (OPT), anterior pretectal nucleus (APT), posterior pretectal nucleus (PPT) and nucleus of the optic tract (NOT) were comprised the pretectal nuclear complex (PNT). Only the APT lacked a retinal input, which was predominantly contralateral in all other nuclei. Our results showed the morphometrical and stereological features of a bat species for the first time.

## Introduction

The order Chiroptera is the second most diverse taxon among the class Mammalia, comprising approximately 1,100 living species (Simmons, 2005). Most bats are nocturnal and usually use echolocation to navigate in the dark (Altringham, 2011). Additionally, most species of bats have an inconspicuous pair of eyes (Altringham and Fenton, 2003), which leads to questions about the functional significance of the visual system in these animals. For example, which structures in the bat brain are involved visual processing, including visuomotor or multisensory integration? What proportion of these structures is linked to the visual functions?

Interestingly, bats are notably absent from the list of species for which the visual system has been deeply described. Much of the research on the visual pathways has been performed in rodents, in which the general patterns of the retinal projection to the dorsal lateral geniculate nucleus (dLGN), superior colliculus (SC) and accessory optic system (AOS) structures have been described (Sefton and Dreher, 1995; Morin and Blanchard, 1997, 1998; Ling et al., 1998; Major et al., 2003; Horowitz et al., 2004; Gaillard et al., 2013). Functional studies have shown evidence of the importance of visual cues under specific environmental conditions, such as luminosity, foraging behavior, predator avoidance and long-range navigation, in bats (Chase, 1981; Greif et al., 2014; Gutierrez et al., 2014), suggesting congruent functions between echolocation and visual information to mediate goal-directed orienting movements (Hoffmann et al., 2016). These recently identified novelties in bat visual functions have created excitement in the scientific community and generated an interest in better understanding the neural framework that underlies the visual system in bats (Melin et al., 2014; Butz et al., 2015; Scalia et al., 2015). On the other hand, several features of the neural arrangement have been neglected in the bat visual neuroanatomy, e.g., regarding the morphologies of the retinal fibers and varicosities that project to a given downstream region (see Sherman and Guillery, 2011) because slight morphological variations in retinal fibers can dramatically alter visual functions (Gauvain and Murphy, 2015).

Fruit-eating bats use echolocation to segregate fruits from vegetation (Kalko and Condon, 1998; Thies et al., 1998); however, fruit in tree branches may produce a very confusing background that is difficult to differentiate using echolocation only, which makes the use of vision critical for accessing food (Korine and Kalko, 2005; Gutierrez et al., 2014; Hoffmann et al., 2016). Recent studies have suggested that several echolocating bats have color vision due the presence of two cone opsins and cone photoreceptors in some species (Feller et al., 2009; Zhao et al., 2009; Melin et al., 2014; Gutierrez et al., 2018). Additionally, a laminar segregation of the retinal fibers in the dLGN is relatively clear in pteropodid bats (Cotter, 1981; formerly classified as Megachiropterans). This laminar segregation is present in species that are heavily reliant on visual cues for their normal behavior, such as carnivores, primates, tree shrews and flying foxes (e.g., Kaas et al., 1978; Dreher, 1986; Casagrande and Norton, 1991; Garey et al., 1991; Rosa et al., 1996; Ichida et al., 2000; Lyon et al., 2003; for reviews).

The present study reports projections from the retina to the primary visual system and pretectal complex in A*rtibeus planirostris* (Chiroptera, Phyllostomidae) using the cholera toxin B subunit (CTb). The CTb is considered the most sensitive technique for mapping retinal projections into the brain (Angelucci et al., 1996; Gaillard et al., 2013; Morin and Studholme, 2014). To date, the pattern of retinal projections in bats is usually identified according to axonal transport of WGA-HRP or unconjugated HRP or via less sensitive methods that reveal the retinal projections (Reimer, 1989; Thiele et al., 1991). On the other hand, a modern description of the retinal projection using CTb as a tracer was recently performed in another Phillostomid bat, the short-tailed fruit bat (*Carollia perspicillata*) and extensively compared with retinal projections in mice (Scalia et al., 2015).

**Figure 1:**
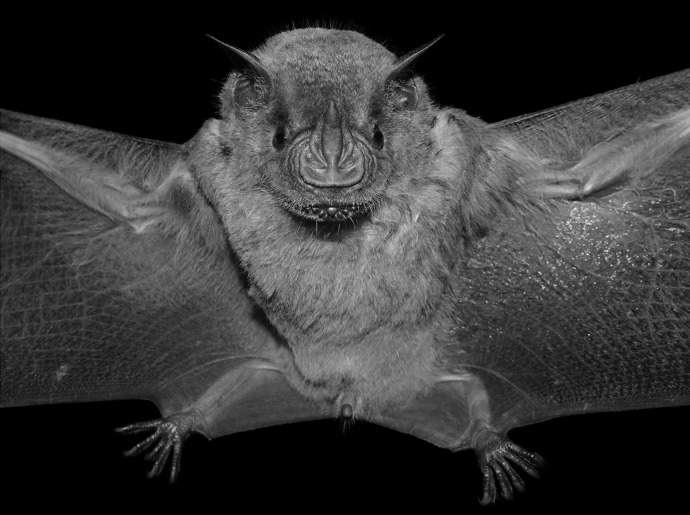
*Artibeus planirostris* (Photo/Image courtesy of Frederico Horie Silva).

*Artibeus planirostris* is a relatively large bat with a total length ranging from 7.5 cm to 11 cm and body mass between 39 g and 69 g (Barquez et al., 1999). The external cranial measurements of *A. planirostris* reveal that it has small and frontalized eyes (8 mm in interorbital width) despite the distance between the eyes exceeding the distance between the eye and end of the muzzle. *A. planirostris* is a fruit-eating bat that is widely distributed throughout tropical lowland areas of South America (Hollis, 2005) and is a common species in many regions of Brazil (e.g., Barros et al., 2017). This species inhabits different types of rain and dry forests and roosts in trees (Hollis, 2005). Although it was recorded at different times during the night, *A. planirostris* is most active in the early evening, especially in the second and third hour after sunset (Bernard, 2002; Figure 1).

Understandably, there is great interest in analyzing the auditory system of bats because bats are notable for their echolocation ability. As opposed to visual imaging, a biosonar image of spatial object properties is a challenge for the auditory system because the sensory epithelium is not arranged along space axes. For echolocating bats, the object width is encoded by the amplitude of its echo (echo intensity) as well as by the naturally covarying spread of the angles of incidence from which the echoes impinge on the bat’s ears (sonar aperture; Heinrich et al., 2011). Thus, bats are able to detect changes in object width according to the absence of intensity of echo cues. On the other hand, several studies have focused on the bat visual system and have provided evidence of vision being used by bats for diurnal navigation (Layne, 1967), pattern discrimination (Suthers et al., 1969), homing (William and Williams, 1970), escape behavior (Chase, 1983), obstacle avoidance (Orbach and Fenton, 2010), locating roosting sites (Ruczyński et al., 2011) and foraging behavior (Gutierrez et al., 2014). Interestingly, there are few detailed reports on the morphologies of the visual structures in these microphthalmic flying mammals (Cotter and Pierson Pentney, 1979; Cotter, 1985; Covey et al., 1987; Reimer, 1989; Scalia et al., 2015). Further, neither morphometric nor stereological information have been collected from bats on this subject to date. The present investigation was conducted to obtain a complete description of the bat retinorecipient brain regions with stereological measures, comparing them with patterns identified in others species. In summary, this study may provide clues about the evolution of the complex visual system in bats.

## Materials and Methods

### Animals and Housing

Ten *A. planirostris* adult males (body weight range, 40–47 g) captured at the campus of the Federal University of Rio Grande do Norte (UFRN), Natal, Northeast Brazil were used in this study as authorized by the Chico Mendes Institute for Biodiversity Conservation (ICMBio Register SISBIO N° 25233-2). We captured bats using three nylon Ecotone^®^ mist nets with dimensions of 3 m × 12 and mesh size of 19 × 19 mm. The nets were opened after sunset and remained exposed for two consecutive hours. The animals were housed at Biosciences Center, UFRN in 0.70 m × 0.50 m × 0.35 m cages, which included 0.15 m × 0.13 m × 0.29 m nest boxes. The individuals were exposed to controlled light, temperature and humidity, with food and water freely available. All experimental procedures strictly followed the rules established by the Ethics Committee on Animal Use (CEUA) of UFRN and were approved by this committee (protocol number 009/2012).

### Eye Injections, Perfusions and Section Collection

Animals were anesthetized with an intramuscular injection of ketamine (5 mg/Kg; Agener), xylazine (0.5 mg/kg; Rhobigarma), diazepam (0.5 mg/Kg; Compaz) and tramadol hydrochloride (5 mg/kg; Cristália). Following topical applications of tetracaine hydrochloride (Allergan) to the cornea, bats were given a unilateral eye injection (left eye) of an aqueous solution that included 15 μl of 5% of the B subunit of cholera toxin (CTb, List Biological Laboratories, Inc., Campbell, CA, USA) in 10% dimethyl sulfoxide (DMSO). This solution was injected into the vitreous humor using a 30-gauge needle catheter attached to a micropump, which pushed the solution at a rate of 0.8 μl/min. To minimize the reflux and spread of the tracer to the extraocular muscles, the needle was left on the site until 15 min post-injection and then withdrawn. To avoid post-operatory local infection, the ocular surface was cleaned with saline during the surgical procedure. Then, the ocular surface was washed with saline and an antibiotic ointment was topically applied. Five days post-injection, bats were reanesthetized with the same anesthetic and perfused transcardially with 150 ml of phosphate-buffered saline, pH 7.4, containing 500 UI heparin (Liquemine, Roche, Brazil), followed by 300 ml of 4% paraformaldehyde in 0.1 M phosphate buffer (PB), pH 7.4. The brain was removed and, after postfixation in the same fixative for 2 h, were cut serially into coronal 30 μm sections using a freezing microtome. The sections were collected individually and placed into a series of six jars filled with PB for subsequent staining. Thus, the anteroposterior interval among sections stained for CTb or thionin was 180 μm.

### Nissl Staining and Immunohistochemistry

Sections from one series were immediately mounted onto electrostatic glass (Fisherbrand) and were then Nissl stained with thionin to visualize the cytoarchitectonic delimitation of the neuronal groups. Sections from another series were submitted to immunohistochemistry to reveal CTb. All of the immunohistochemistry procedures were performed at room temperature. The sections, previously submitted to pre-treatment with hydrogen peroxide (H_2_O_2_), were free floating incubated in a blocking solution containing bovine serum albumin (BSA); diluted in 5% Triton X-100 for 1 h; and incubated for 18–24 h with the primary antiserum, a goat anti-CTb IgG (List Biological Labs, Campbell, CA, USA; RRID: AB_10013220) diluted 1:1,000 in solution containing 2% BSA, 0.4% Triton X-100 and 0.1 M PB, pH 7.4. The sections were then incubated with a biotinylated secondary antiserum (donkey anti-goat IgG, JacksonLabs, Westgrove, PA, USA) diluted 1:1,000 for 90 min. The sections were subsequently incubated with an avidin–biotin–peroxidase solution (ABC Elite kit, Vector Labs, Burlingame, CA, USA) for 90 min in 0.4% Triton X-100 NaCl. The sections were then reacted for peroxidase in a solution of diaminobenzidine tetrahydrochloride (DAB, Sigma, St Louis, MO, USA) and 0.01% H_2_O_2_ in 0.1 M PB, pH 7.4. The sections were washed with 0.1 M PB, pH 7.4 (5 × 5 min) between each step and at the end of the procedure. The sections were then allowed to dry, dehydrated through a graded alcohol series, cleared in xylene, and cover-slipped with the neutral mounting medium ERV-MOUNT (EasyPath). Specificity tests were performed based on omission of the primary or secondary antibodies in some sections. In all cases immunolabeling was completely abolished. Furthermore, as a control for the possibility of transsynaptic labeling, the occipital lobes of one animal were sectioned and processed for CTb immunostaining. The visual cortex of these animals was examined to verify the presence of CTb. As a result, immunolabeling was completely abolished.

### Digital Photography

The sections were examined under bright field illumination on a Nikon microscope (Nikon Eclipse/Ni-U), and digital images of representative sections were taken by a video digital camera (Nikon DS-Ri1). The images were minimally processed for brightness and contrast, and drawings were made using Canvas 12 with the aid of The Rat Brain in Stereotaxic Coordinates (Paxinos and Watson, 2007) and Forebrain Atlas of the Short-tailed Fruit Bat, *Carollia perspicillata* (Scalia et al., 2013).

### Relative Optical Density

For quantitative analysis, all images were obtained under brightfield illumination at a fixed intensity for each of the rostrocaudal levels per individual. The resulting brightfield images were 3840 × 3072 pixel, with a resolution of 0.59 pixel/μm (with a 4× objective). Relative Optical Density (ROD) analysis was performed using Image J software (Version 1.49i, NIH). The images were converted to gray scale images (8-bit). The images were then binarized and the contrast was adjusted to 100%. After this procedure, the images only had two values, which were expressed on a histogram: zero, which corresponded to black, and 255, which corresponded to white. Finally, the program provided the number of black pixels in the sampled areas (Santos et al., 2013). The mean gray value of each sampled area was measured using a square (0.3 × 0.3 mm) in the area of interest (AOI). For dLGN and SC, eight squares were sampled, four in each layer, and for each Pretectal Nuclear Complex (PNT) nucleus, one square was sampled on well-defined DAB stained sections throughout the rostrocaudal levels. The mean gray values were calculated. The medium number of black pixels in the target area was subtracted from the median values of a control region (areas that should not have specific CTb staining). The value of the optical density (OD) of the AOI was related to the mean of the black pixel values in the target area and the mean background value calculated the formula: ROD = [(OD AOI − OD background)/(OD AOI + OD background)]. The data from each target area are expressed as the mean of pixels in the AOI. Manual selection of the chromogen that stained positively for DAB was performed (Cuesta et al., 2013). All results are expressed as the mean and standard deviation of the mean (SD).

### Stereology and Morphometry

The Cavalieri principle (Howard and Reed, 2005) was used to estimate the nuclear volume in the primary visual system. We selected 7–9 sections of the dLGN, 3–4 sections of the PNT and seven sections of the SC from Nissl-stained coronal sections with had a 30 μm thickness and were obtained from five animals using systematic and uniform random sampling (SURS; Gundersen et al., 1999) for each area. These sections were analyzed under a 5× microscope objective with the aid of MBF microsystem Stereo Investigator Software coupled to the Zeiss Imager M.2 Microscope with ApoTome.2. The following formula was used to estimate the nuclear volume: V = Σp. a/p. t. F^−1^. The error coefficient (EC) for the volume estimation according to the Cavalieri Principle was calculated according to the formula: EC = √Var [total]/∑p (see Gundersen et al., 1999 for EC measurement details).

For the cellular area measurement, we used all of the Nissl stained coronal sections that were found in the regions of interest from five animal brains, and we obtained the cellular profile of all of the extensions of the nuclei. The sections were analyzed under a 20× microscope objective with the aid of NIS ELEMENTS AR software coupled to a Nikon Ni-U Microscope. We selected 10 cells per section of the dLGN, five in the outer layer and five in the inner layer, totaling 360 measured cells; 10 cells per section of the SC, five cells in zonal stratum (ZS) layer and five cells in superficial gray superior colliculus (SGS) layer, totaling 500 measured cells; and finally, 10 cells per section of each nucleus of the NPT, totaling 120 cells for nucleus of the optic tract (NOT), 120 for olivary pretectal nucleus (OPT), 110 for PMT and 80 for posterior pretectal nucleus (PPT).

### Statistical Analysis

The General Linear Mixed Model (GLMM) was used to analyze the influence of the animal, section and layer (fixed factors) on the variation of the cellular area (response variable) and the influence of the animal, section, layer and hemisphere (fixed factors) on the variation of the ROD values (response variable). These model analyses were performed separately for each nucleus. Another GLMM was performed to verify differences among nuclei (fixed factor) with respect to their volume (response variable). Pair wise comparisons were performed using Bonferroni’s *post hoc* test. A significance level of 5% or less was considered for all tests. The statistical software IBM SPSS Statistics 21 was used for all data analyses.

## Results

We used stereological methods to estimate the volume of the studied nuclei as well as the average of the neuronal areas to distinguish nuclei and their subdivisions. Additionally, to differentiate the density of the retinal fibers among the investigated nuclei, we used the ROD. Finally, the quality of fibers was accessed from high magnification photomicrographs of the sampled areas. The rostrocaudal length of the encephalon of *A. planirostris* from the olfactory bulb to the bulb-spinal transition was approximately 17.65 mm. Nissl-stained sections helped us to establish the anatomical boundaries of the nuclei and cytoarchitecture. To provide a comparative morphometric analysis between neurons in the various subdivisions of the studied nuclei, we considered large neurons to be those with areas ranging between 150 μm^2^ and 100 μm^2^. Medium neurons were considered to be those with areas between 99 μm^2^ and 50 μm^2^, and small neurons were considered to be those with areas under 50 μm^2^.

### Cytoarchitectonic, Morphometrical and Stereological Analysis

**Figure 2:**
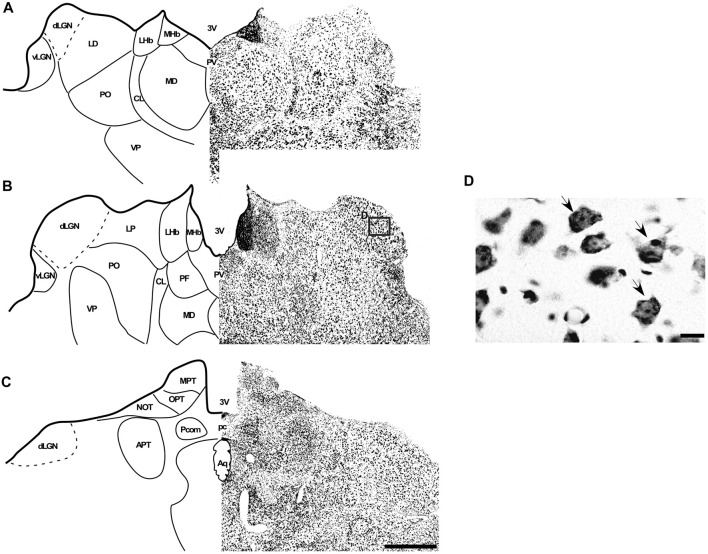
Photomicrographs of the brain sections of flat-faced fruit-eating bat showing the dLGN in bright field stained by Nissl technique at rostral, middle and caudal levels (right) and drawings (left; **A–C**), respectively. The boxed area in **(B)** are shown in high magnification in (D), illustrating the detailed morphology of the cells in the dLGN. Black arrows indicating rounded shape neurons in the dLGN. Scale bar 100 μm **(A–C)** and 10 μm **(D)**. Abbreviations: see list.

**Figure 3:**
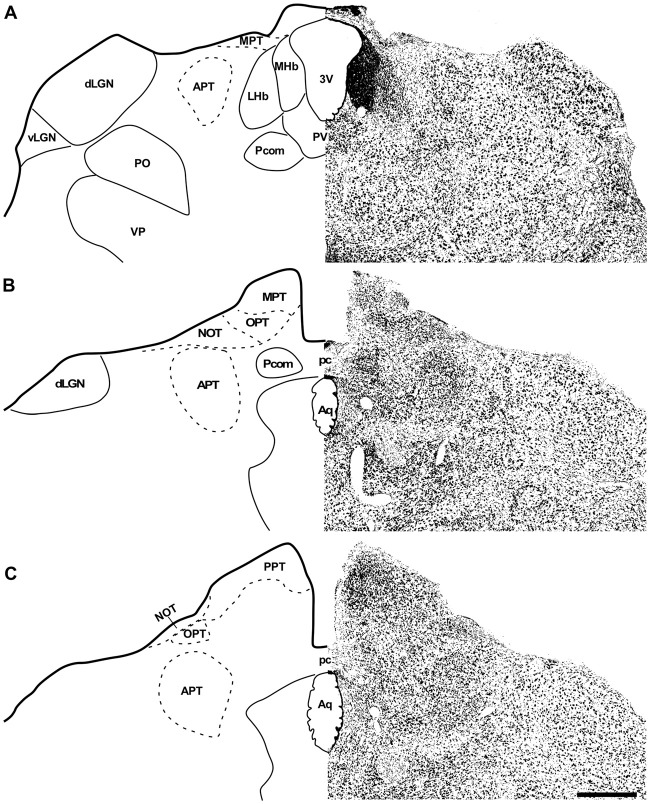
Photomicrographs of the brain sections of flat-faced fruit-eating bat showing the PNT in bright field stained by Nissl technique at rostral, middle and caudal levels (right) and drawings (left; **A–C**), respectively. Scale bar 100 μm **(A–C)**. Abbreviations: see list.

**Figure 4:**
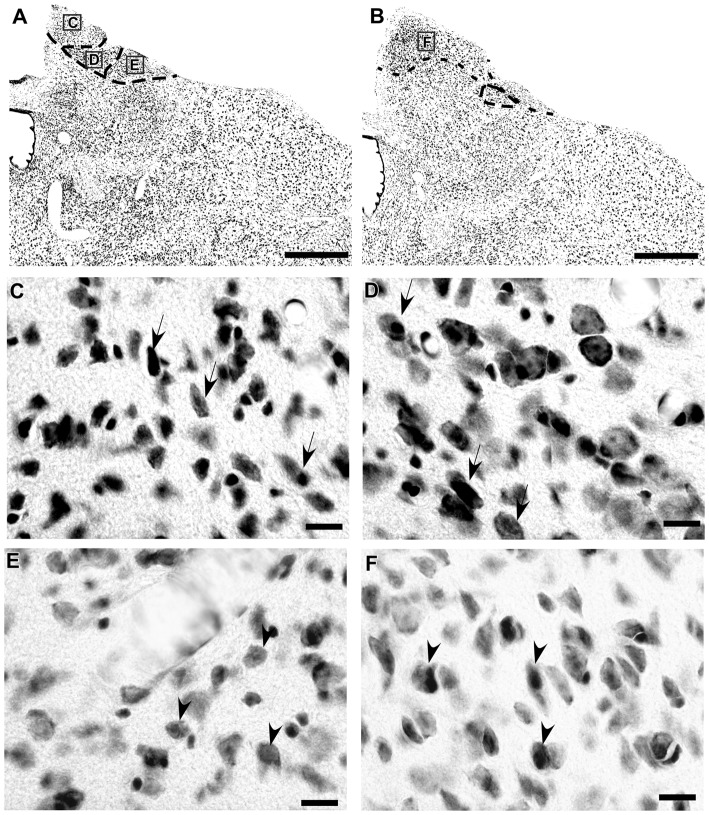
Photomicrographs of the brain sections of flat-faced fruit-eating bat showing the PNT in bright field stained by Nissl technique at middle and caudal levels **(A,B)**. The boxed area in **(A,B)** are shown in high magnification in (C–F), illustrating the detailed morphology of the cells in the MPT, OPT, NOT and PPT respectively. Black arrows indicating elliptical shaped neurons and black arrow heads indicating round shaped neurons. Scale bar 100 μm **(A,B)** and 10 μm **(C–F)**. Abbreviations: see list.

**Figure 5:**
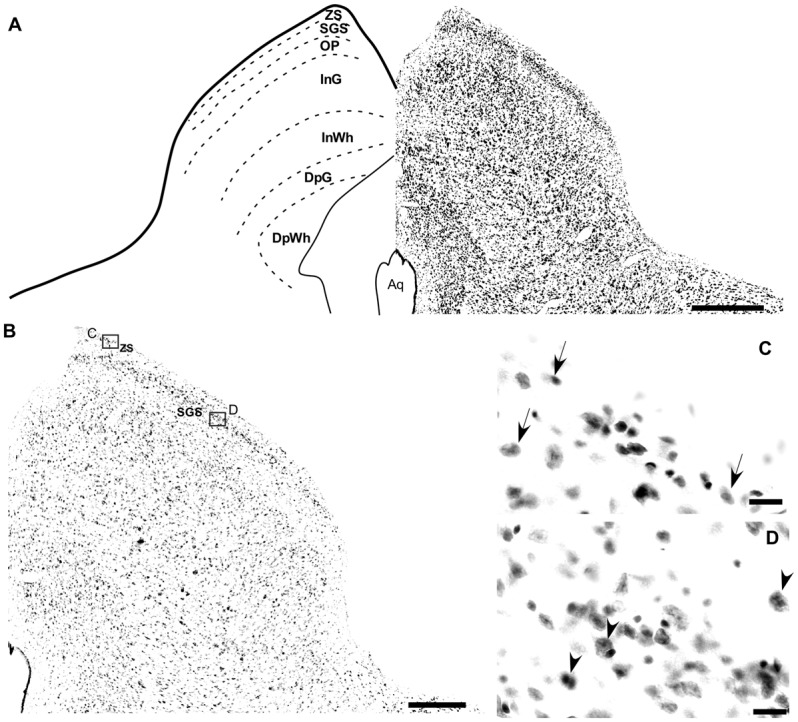
Photomicrographs of the brain sections of flat-faced fruit-eating bat showing the SC in bright field stained by Nissl technique at middle level (right) and drawing (left; **A**) The boxed areas in **(B)** are shown in high magnification in (C,D), illustrating the detailed morphology of the cells in the SC. Black arrows indicating elliptical shaped neurons in the ZS and black arrow heads indicating round shaped neurons in the SGS. Scale bar 100 μm **(A,B)** and 10 μm **(C,D)**. Abbreviations: see list.

In the coronal sections of the brain of *A. planirostris*, the dLGN was easily identified as a cluster of cells in the most dorsal edge of the thalamus in the rostral sections, which lies laterally in the mid- and caudal-levels of the dorsal thalamus (Figures 2A–C). It was not possible to identify differences among layers in the dLGN of *A. planirostris* using Nissl-stained coronal sections of the thalamus, despite the observation of large and round shaped neurons in Nissl preparations being present throughout the nucleus (mean area = 149.1 μm^2^; Figure 2D). In *A. planirostris*, the neurons in the dLGN had the largest average cellular area among the visual nuclei investigated (*p* < 0.001; Table 1). The coronal sections in the midbrain revealed all of the classical nuclei of the PNT. The anterior pretectal nucleus (APT) lied ventrally to the remaining nuclei of the PNT and medial to the dLGN (Figure 3A). Neurons in the APT were medium-sized in area (mean area = 65.1 μm^2^; Table 1) and predominantly round shaped. The APT extended caudally to the level of the posterior commissure (pc; Figures 3B,C). The medial pretectal nucleus (MPT) was observed dorsal to the habenular complex in the rostral sections (Figure 3A) as well as dorsal to the NOT and OPT in the mid sections of the PNT (Figure 3B). The PPT in the caudal sections of the midbrain was visualized to lie dorsal to the OPT at the caudal sections (Figure 3C). Cytoarchitectonic analysis revealed predominantly elliptical shaped neurons in the MPT and OPT (Figures 4A–D). Otherwise, round shaped neurons dominated the microscopic fields in the NOT and PPT (Figures 4E,F). In addition, neurons in the PPT had the largest average area among nuclei in the PNT (mean area = 84.4 μm^2^). Meanwhile, the NOT neurons had the smallest average area among PNT nuclei (mean area = 29.5 μm^2^; Table 1). The SC in *A. planirostris* lies dorsal in the midbrain at the same level of the most caudal OPT section. Analysis of Nissl-stained sections in the midbrain provided unequivocal evidence of seven layers in the SC of *A. planirostris* (Figures 5A,B). The ZS was the most superficial layer in the SC, with small (mean area = 32.2 μm^2^) and elliptical shaped neurons (Figure 5C). Nissl preparation and posterior morphometrical analyses revealed round shaped neurons in the superficial gray layer (SGS; Figure 5D), with neurons that were significantly larger in area (mean area = 55.8 μm^2^) than those in the ZS (*p* < 0.001; Table 1). Together, ZS and SGS represent the most superficial layers in *A. planirostris’* SC, which are collectively the main target of the retinal projection in the SC of this species.

**Table 1:** Morphometrical and stereological analysis.

	Dorsal lateral geniculate nucleus				Superior colliculus		
Animal^a^/Weight (g)	Superficial layer Mean ± SD (μm^2^)	Deep layer Mean ± SD (μm^2^)	Volume (μm^3^) CE = 4.5%	Animal^a^/Weight (g)	ZS Mean ± SD (μm^2^)	CGS Mean ± SD (μm^2^)	Volume (μm^3^) CE = 4.9%
B2/45.5	140.9 (±32.7)	165.3 (±33.0)	1.20	B5/46.0	39.1 (±13.7)	66.3 (±21.2)	6.20
B4/40.0	137.6 (±32.6)	139.0 (±31.3)	1.08	B7/41.0	36.0 (±12.5)	58.2 (±19.2)	6.01
B8/39.5	110.7 (±37.6)	114.0 (±33.7)	0.91	B8/39.5	29.8 (±9.6)	48.7 (±15.3)	5.75
B9/41.0	199.2 (±128.7)	195.3 (±79.9)	1.14	B9/41.0	25.4 (±8.1)	49.8 (±13.0)	6.39
B10/40.5	136.4 (±30.4)	139.0 (±40.5)	1.10	B10/40.5	31.1 (±12.0)	56.3 (±16.2)	6.59
**Mean**	**146.5 (±73.2)**	**151.8 (±55.8)**	**1.08**	**Mean**	**32.2 (±12.2)**	**55.8 (±18.2)**	**6.19**
	**NS**				***p* < 0.001**		*p < 0.001
**Pretectal nuclear complex**							
**Animal^a^/Weight (g)**	**MPT Mean ± SD (μm^2^)**	**OPT Mean ± SD (μm^2^)**	**NOT Mean ± SD (μm^2^)**	**PPT Mean ± SD (μm^2^)**	**Volume (μm^3^) CE = 5.7%**		
B1/40.5	57.4 (±25.6)	69.1 (±24.8)	24.9 (±9.3)	107.4 (±35.3)	1.02		
B7/41.0	50.4 (±44.7)	69.6 (±31.0)	26.2 (±10.6)	89.5 (±32.2)	0.95		
B8/39.5	57.5 (±30.1)	57.6 (±18.2)	55.4 (±13.4)	35.8 (±6.4)	0.81		
B9/41.0	35.7 (±15.6)	58.4 (±22.8)	26.3 (±21.0)	76.1 (±27.8)	1.17		
B10/40.5	42.3 (±18.0)	64.0 (±25.0)	33.7 (±15.6)	84.8 (±32.0)	0.79		
**Mean**	**46.8 (±30.2)**	**64.7 (±25.7)**	**29.5 (±16.6)**	**84.4 (±36.5)**	**0.95**		
	a**	b**	c**	d**			

Mean values of cellular area and standard deviations in the investigated cases per retinorecipient subdivision and total estimated volume of dorsal lateral geniculate nucleus (dLGN), superior colliculus (SC) and pretectal nuclear complex (PNT). *Related to dLGN and PNT; **Different letters represent significant differences among PNT nuclei (Bonferroni *post hoc* test, *p* < 0.001). Animal^a^: B. bat. NS: non significant. CE: coefficient of error.

Stereology of the primary visual nuclei to estimate volumes was performed. The SC (6.19 mm^3^) volume in *A. planirostris* was higher (*p* < 0.005) than those of the dLGN (1.08 mm^3^) and PNT (0.95 mm^3^). On the other hand, no significant difference was detected between the LGN and PNT (*p* < 0.001; Table 1).

### Retinal Projections

The retinal fibers in the primary visual nuclei of *A. planirostris* were revealed according to CTb anterograde transport after an intraocular injection. The distinctive features of the fibers in the terminal field were clearly demonstrated in the dLGN, PNT and SC, suggesting that the CTb accumulated in the arbors of the optic axons prior to sacrifice. The retinal projection in *A. planirostris* was predominantly contralateral to the eye injections. Normally, stained CTb fibers densely and completely fill nuclei, which are generally opaque, impairing qualitative analysis of retinal fibers. Qualitative evaluation was performed when discrete preterminal axons and boutons could only be resolved on the projection that appeared lighter, as on the fringes of densely innervated areas and on the ipsilateral side. In general, three types of terminals were identified in *A. planirostris’* visual primary nuclei: (1) type R1-like terminals, which consisted of large, elliptical varicosities along the length of the caliber axons, and type R2-like terminals, which consisted of medium and small varicosities forming rosette-like clusters of boutons; (2) string-like configurations, which consisted of axons collaterally studded with boutons of various sizes; and (3) simple *en passant* varicosities and terminal swellings, which were present in poorly branched fibers decorated with a varicosity at the end of fiber.

**Figure 6:**
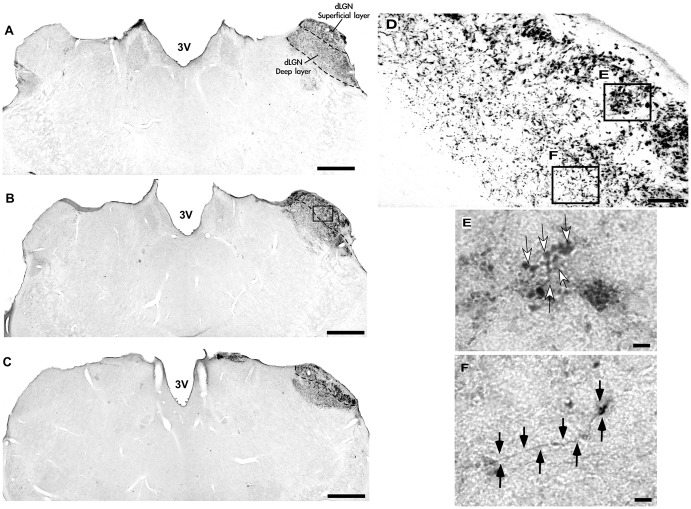
Photomicrographs of the dLGN coronal sections of flat-faced fruit-eating bat at rostral **(A)**, middle **(B)** and caudal **(C)** levels, illustrating the distribution pattern of retinal projections in the ipsi and contralateral sides. The boxed areas in **(D)** are shown in high magnification in (E,F) respectively, illustrating the detailed morphology of the retinal axons in the superficial **(E)**, and deep layers in the contralateral side of the dLGN. White arrows indicating R1and R2-like terminals in the superficial layer **(E)**, and black arrows indicating simple endings **(F)**. Scale bar 100 μm **(A–D)** and 10 μm **(E,F)**. Abbreviations: see list.

**Figure 7:**
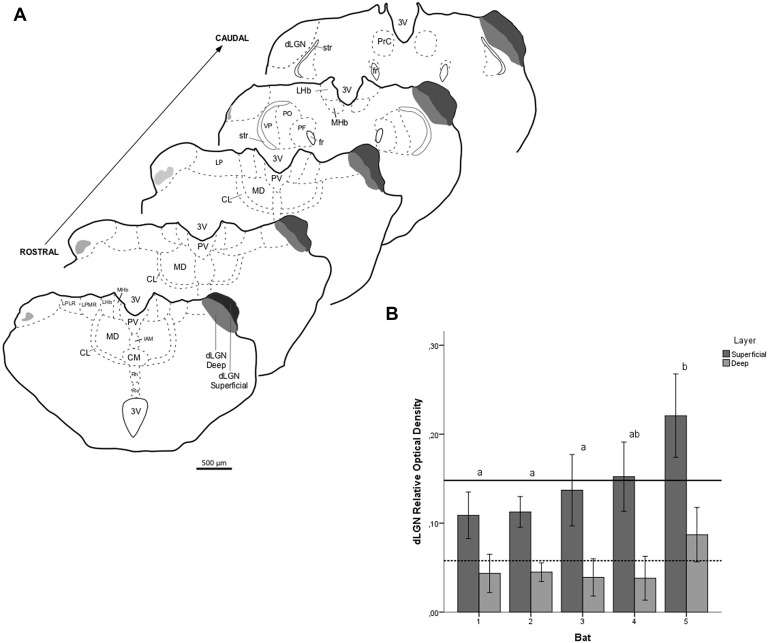
Relative optical density (ROD) values in the dLGN layers of flat-faced fruit-eating bat (*n* = 5). To compare across layers, the ROD in each layer was schematically displayed as levels of gray in the drawings **(A)** through rostrocaudal length. The bars represent the means (± standard error) of the mean ROD values of individual animals by layer **(B)**. The dashed and solid lines represent the overall mean of ROD for the deep and superficial layers, respectively. General Linear Mixed Model (GLMM) revealed ROD significant difference between the layers analyzed (*F*_(1,109)_ = 60.6, *p* < 0.001). Different letters represent significant individual differences (Bonferroni *post hoc* test, *p* < 0.05). Scale bar 500 μm in **(A)**. Abbreviations: see list.

**Figure 8:**
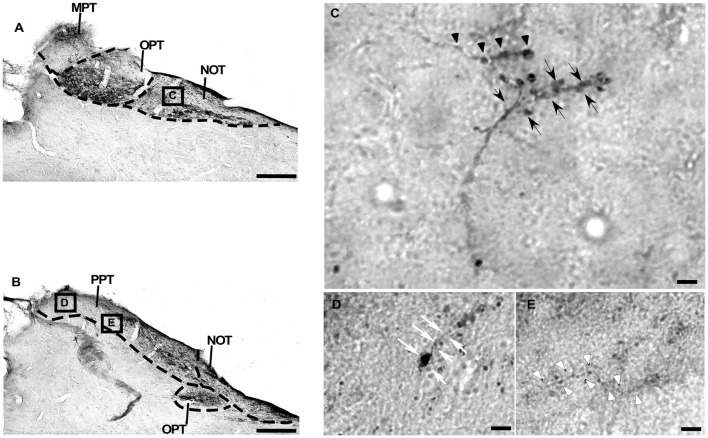
Photomicrographs of the PNT coronal sections of flat-faced fruit-eating bat at middle **(A)** and caudal **(B)** levels, illustrating the distribution pattern of retinal projections. The boxed areas in **(A,B)** are shown in high magnification in (C–E), respectively. The boxed area in **(A)** is shown in high magnification in (C), illustrating the detailed morphology of the retinal axons in the NOT. The boxed areas in **(B)** are shown in high magnification in (D,E), respectively, illustrating the detailed morphology of retinal axons in the PPT **(D,E)**. Black arrows indicating R2-like terminals; Black arrow heads indicating R1 like-terminals; White arrows indicating simple ending-like terminals; and White arrow heads indicating string-like terminals. Scale bar 100 μm **(A,B)** and 10 μm **(C–E)**. Abbreviations: see list.

**Figure 9:**
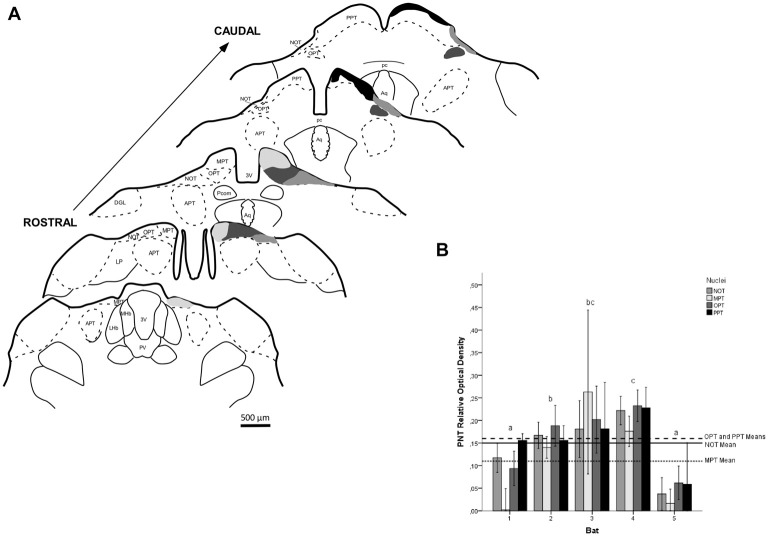
ROD values in the PNT of flat-faced fruit-eating bat (*n* = 5). To compare among nuclei, the ROD in each nucleus was schematically displayed as levels of gray in the drawings **(A)** through rostrocaudal length. The bars represent the means (± standard error) of the mean ROD values of individual animals by nuclei **(B)**. GLMM did not reveal ROD significant difference among nuclei analyzed (*F*_(3,78)_ = 1.61, *p* = 0.193). Different letters represent significant individual differences (Bonferroni *post hoc* test, *p* < 0.05). Scale bar 500 μm in **(A)**. Abbreviations: see list.

**Figure 10:**
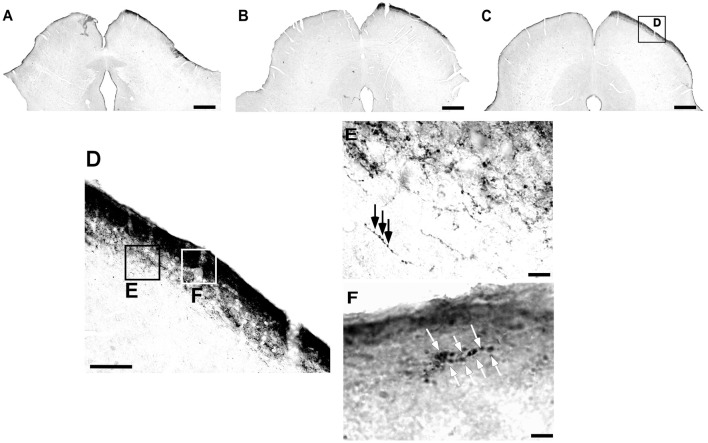
Photomicrographs of the SC coronal sections of flat-faced fruit-eating bat at rostral **(A)**, middle **(B)** and caudal **(C)** levels, illustrating the distribution pattern of retinal projections in the ipsi and contralateral sides. The boxed areas in **(D)** are shown in high magnification in E (SGS) and F (ZS), respectively. White arrows indicate R2 and R1-like terminals; and Black arrows indicating string-like terminals. Scale bar 100 μm **(A–D)** and 10 μm **(E,F)**. Abbreviations: see list.

**Figure 11:**
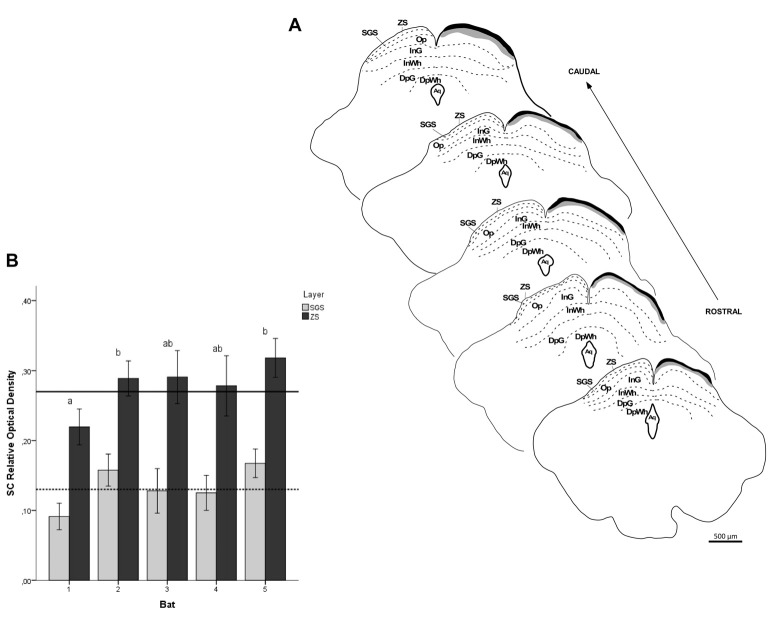
ROD values in the SC of flat-faced fruit-eating bat (*n* = 5). To compare across layers, the ROD in each layer was schematically displayed as levels of gray in the drawings **(A)** through rostrocaudal length. The bars represent the means (± standard error) of the mean ROD values of individual animals by layer **(B)**. The dashed and solid lines represent the overall mean of ROD for the SGS and ZS layers, respectively. GLMM revealed ROD significant difference between the layers analyzed (*F*_(1,117)_ = 117.4, *p* < 0.001). Different letters represent significant individual differences (Bonferroni *post hoc* test, *p* < 0.05). Scale bar 500 μm in **(A)**. Abbreviations: see list.

The retinal projection to the dLGN of *A. planirostris* was completely contralateral (Figures 6A–C). In addition, the dLGN appeared to be divided into two layers in accordance with the types of fibers (Figures 6D–F). The superficial division of the dLGN had predominantly R1/R2-like terminals (Figure 6E). On the other hand, simple en passant fibers were predominantly observed in the deep layer of the nucleus (Figure 6F). Consistent with that observation, layering suggested by the type fibers distribution analysis in the dLGN was confirmed by use of the ROD approach, in which the LGN also presented significant differences in ROD values between these sectors of the nucleus (*p* < 0.001; Figure 7). The PNT nuclei have shown retinal fibers with a contralateral predominance in all nuclei of the complex, except APT, in which no fibers were observed throughout rostrocaudal extension (Figures 8A,B). Although, slight differences were resolved according to ROD analysis, there were no significant differences among retinorecipient areas in the PNT, suggesting a relatively homogeneous retinal fiber distribution in this nuclear complex (Figure 9). High magnification analysis of the fibers in the PNT showed that simple end-like fibers, as well as string-like fibers, were homogeneously distributed throughout the MPT, OPT and PPT nuclei (Figures 8D,E). On the other hand, R2-like terminals were seen predominantly in the NOT, despite few R1-like terminals have been visualized in this area (Figure 8C). Coronal brain sections of *A. planirostris* were also used to revealed that the pattern of retinal fibers in the SC had an exclusively contralateral distribution (Figures 10A–C). Retinal fibers were restricted to the most superficial collicular layer ZS and immediately deeper layer SGS in the SC of *A. planirostris* (Figure 10D). The ROD of these two retinorecipient collicular layers had significantly higher ROD values in the ZS compared to those in the SGS layer (*p* < 0.001; Figure 11). Furthermore, high magnification of the retinal fibers inside the SC showed that R1 and R2-like terminals were predominantly distributed in the ZS layer (Figure 10F), differing from that observed in the SGS layer, which predominantly contained string-like terminals (Figure 10E).

## Discussion

The present study is the first to reveal subcortical projections of the retina to the primary visual structures in the brain of *A. planirostris* and provides a detailed anatomical description of these visual nuclei using reliable quantitative tools, such as stereological and morphometrical analysis, as well as the ROD of the retinal projections. Surprisingly, retinal projections that usually target the three nuclei of the rostral midbrain: DTN, MTN and LTN of the AOS (Weber, 1985; Zhang and Hoffmann, 1993) and are functionally involved in mediating visuomotor reflex such as underling the generation of optokinetic nystagmus (Precht and Strata, 1980; Hoffmann and Distler, 1986), as well as visual regulation of the vestibular ocular reflex (Ito et al., 1979) seem to be absent in *A. planirostris*.

### Dorsal Lateral Geniculate Nucleus

Cytoarchitecturally, the dLGN in *A. planirostris* does not appear to be laminated, even using a morphometrical approach in which no significant difference was observed among neuronal areas. A similar pattern was observed in the genus *Rhinolophus* (Niimi et al., 1963), *Myotis lucifugus* (Pentney and Cotter, 1976), and *Carolia perspicilata* (Scalia et al., 2015). Larger (mean area = 149.1 μm^2^) cells were observed in the dLGN compared to those in the SC and PNT of *A. planirostris*. A previous study in the gray-headed flying fox (*Pteropus poliocephalus*) revealed small neurons (106 μm^2^) in the dLGN (Ichida et al., 2000) compared to those in the dLGN in *A. planirostris*. Scalia et al. (2015) have performed an extensive study of subcortical visual centers in the bat *Carolia perspicilata*. These authors have reported neurons as being larger in the dLGN, despite no quantitative data being provided. Finally, we used stereological methods to estimate the volume of the dLGN (1.08 mm^3^) of *A. planirostris*. As a result, we attest that the dLGN in *A. planirostris* is surprisingly larger than that reported in the Nile grass rat (*Arvicanthis niloticus*; 0.55 mm^3^), mice (0.32 mm^3^) and rats (0.95 mm^3^; Gaillard et al., 2013), suggesting a well-developed dLGN in bat species.

The retinal fibers that travel to the dLGN are substantially contralateral in *A. planirostris*, with a slight ipsilateral component at the deep layer revealed by ROD values. The projection from the retina distributed uniformly in the rostrocaudal extension of the dLGN, and contrary to morphometrical analysis, a ROD approach revealed superficial and deep layers in the dLGN. Previous studies in bat, using fiber degeneration after eye removal or an intraocular CTb injection have shown lateral and medial layers in the dLGN, even though no quantitative approach has been performed (Cotter and Pierson Pentney, 1979; Cotter, 1985; Covey et al., 1987). In addition, three layers were identified in *Pteropus poliocephalus* (Ichida et al., 2000; Manger and Rosa, 2005) and six layers are classically described in primates (Fitzpatrick et al., 1980; Callaway, 2005). Taken together, these findings suggest that the visual system in bats, including *A. planrostris*, might function as a series of parallel pathways, conveying different aspects of the visual information, similar to that seen in upper vertebrates, which to a great extent orient their behavior by sight. In contrast, a recent study in *Carolia perspicilata* showed a relatively uniform distribution of retinal fibers only in the posterior half of the dLGN, which seem to be largely clustered at various loci into islands or nests in the middle of neurophils (Scalia et al., 2015).

At high magnification, the appearance of retino-dLGN axon arbors in *A. planirostris* resembles that of other terminal specializations of retinofugal axons in the mediodorsal thalamic nucleus, Zona Incerta of the rock cavy and suprachiasmatic hypothalamic nucleus of the *A. planirostris* (do Nascimento et al., 2010; de Góis Morais et al., 2014; Santana et al., 2018). R1/R2 types terminals observed in the superficial layer of the dLGN in the *A. planirostris* have a morphology dramatically distinct, by virtue of their large-sized boutons and rosette-like configuration, from terminals found in the deep layer of the dLGN. These morphological diversities of the endings observed in the superficial and deep layers of the dLGN of *A. planirostris* could be due to source-specific factors, such as subpopulations of retinal ganglion cells, forming cell-specific terminals in the dLGN. Additionally, these distinct morphologies of retinal afferents in the dLGN suggest a differential influence on postsynaptic cells (Sherman, 2005; Petrof and Sherman, 2013) as well as a functional dichotomy in the dLGN.

### Pretectal Nuclear Complex

The dorsocaudal region of the diencephalon, just rostral to the midbrain, develop from prosomere 3 of the diencephalic embryonic vesicle (Puelles and Rubenstein, 1993; Puelles, 1995) and contains an important structure which forms a landmark in the brain of all vertebrates, the pc. The nuclei groups around the pc form an often poorly defined anatomical region named pretectum because of its relative position just anterior to optic tectum (SC in mammals). Interestingly, the retinorecipient pretectal regions are the best characterized nuclei in the pretectum. The PNT in *A. planirostris* is composed of five classical subdivisions that were previously described in mammals: APT, NOT, OPT, MPT and PPT (Kaas and Huerta, 1988; Matteau et al., 2003; Scalia et al., 2015), even though the PPT in the present work has been displaced caudally, resembling topographically to the PPT in the *Carollia perspicillata* (Scalia et al., 2015) and similar to the tectal gray, which have been shown in recent rodent atlases (Puelles et al., 2012).

Despite their diverse nuclear organization, PNT nuclei have been reported in bats (Cotter and Pierson Pentney, 1979), Nile grass rats (*Arvicanthis niloticus*; Gaillard et al., 2013), mice (Morin and Studholme, 2014), and hamsters (Morin and Blanchard, 1998). The APT and PPT are apparently the largest structures in the PNT complex in *A. planirostris*. In fact, this feature corroborates that previously described in *Carollia perspicillata* (Scalia et al., 2015). Furthermore, PPT apparently contains the largest neurons among pretectal nuclei in *Carollia perspicillata*, as in *A. planirostris*. In addition, the neurons in the APT of *A. planirostris* are medium-sized compared to the neurons in the PPT based on morphometrical measurements, as observed in the APT of *Carollia perspicillata* (Scalia et al., 2015). On the other hand, NOT neurons are described as being large in rats (Scalia and Arango, 1979), Nile grass rats (Gaillard et al., 2013), and *Carollia perspicillata* (Scalia et al., 2015), but seem to be the smallest neurons among pretectal nuclei in *A. planirostris*.

Few reports on subcortical visual structures have used stereology to provide a three-dimensional interpretation of these nuclei. In the present work, the PNT was 0.95 mm^3^, indicating a relatively small volume compared to the dLGN and SC. Crish et al. (2006) estimated the volume of the OPT in the naked mole-rat (*Heterocephalus glaber*; 0.01 mm^3^) and mouse (0.03 mm^3^). On the other hand, Gaillard et al. (2013) performed a morphometrical analysis in the PNT of the Nile grass rat (*Arvicanthis niloticus*) and revealed a total rostrocaudal extension of the PNT (1450 μm). Generally, the PNT volume results of *A. planirostris* could not be compared with others species.

The analysis using ROD revealed an uneven retinal projection among nuclei in the PNT. Similarly, high magnification analysis of the retinal fibers throughout the PNT demonstrated both small and large differences in the wiring configuration among nuclei in *A. planirostris*. The ROD measurements note the PPT as the main target of retinal projections in this nuclear complex of *A. planirostris*, followed by OPT, NOT and MPT. It is generally accepted the PPT, OPT, and NOT are involved in the pupillary light reflex and the detection of luminance (Tokunaga et al., 1981; Weber, 1985; Klauer et al., 1990; Zhang and Hoffmann, 1993). Our findings indicate that the retina has denser projections to PPT, OPT, and NOT compare to that one in the MPT. Curiously, R1/R2-like terminals were observed in the entire NOT full length and some parts of the OPT. As expected, no retinal fibers appeared to invade the APT, which is thought to be associated primarily with the somatosensory system and has been implicated in the processing of pain-related information (Reis et al., 2012) and also in the memory functions involved in visual discrimination learning (Thompson, 1979). A distinct retinal fibers distribution has been reported in two bat species, the Indian flying fox (*Pteropus giganteus)* and little brown bat (*Myotis lucifugus*; Cotter and Pierson Pentney, 1979). In *P. giganteus*, the fibers reached all subdivisions of the PTN, except the MPT. On the other hand, fibers from the retina in *Myotis lucifugus* only reached the NOT and OPT. The retinal projection in *A. planirostris* is on superficial cellular edge of the PPT. This pattern of retinal fiber distribution is quite similar to that reported in *Carollia perspicillata* (Scalia et al., 2015).

### Superior Colliculus

The SC in *A. planirostris* is composed of seven distinct layers according to Nissl staining as well as morphometrical analysis. This pattern of cellular organization in the SC seems to be usual among mammalian orders (Kanaseki and Sprague, 1974; Kaas and Huerta, 1988; Zhang and Hoffmann, 1993; Major et al., 2000, 2003; Crish et al., 2006; Nemec et al., 2008; Gaillard et al., 2013; Morin and Studholme, 2014). The stereology performed in this study revealed a relatively large SC in *A. planirostris* (6.19 mm^3^). In the naked mole-rat (*Heterocephalus glaber*), the volume of the SC is 1.41 mm^3^ (Crish et al., 2006). Furthermore, the SC has been shown to be 6.07 mm^3^ in the Nile grass rat (*Arvicanthis niloticus*), 3.00 mm^3^ in mice, and 11.45 mm^3^ in rats (Gaillard et al., 2013). In summary, comparative analysis of the SC in *A. planirostris* indicates the relevance of movement orientation in bats since the SC volume in this species had a similar layering pattern and volume to that of rodents.

The earliest reports, which utilized a functional approach, revealed that superficial layers (ZS, SGS and OP) are related to visual information, while deeper layers (Intermediate gray layer superior colliculus, InG, Intermediate white layer superior colliculus, InWh, Deep gray layer superior colliculus, DpG and Deep white layer superior colliculus, DpWh) are concerned with auditory and somatosensory information (Grantyn and Berthoz, 1985; Meredith and Stein, 1986; Kaas and Huerta, 1988; May, 2006). Remarkably, the present work found that retinal projections were entirely in the contralateral SC in *A. planirostris*. Moreover, retinal fibers were restricted and uniformly distributed throughout the rostrocaudal extension of the ZS and SGS layers in *A. planirostris*. Interestingly, the ZS layer exhibited a significantly higher value of ROD compared to that in the SGS, and R1/R2-type terminals were predominantly observed in the ZS layer. Overall, this morphological arrangement suggests a strong effect of light on cellular activity in these layers of the CS in order to influence movement orientation in *A. planirostris*. In *Carollia perspicillata*, retinal projections to the SC are directed entirely to the contralateral side, but just reach the SGS and do not cover the collicular surface uniformly. They also seem to be weak or absent in the anterior and posterior thirds of the SC (Scalia et al., 2015). The retinal projections in the SC of the large naked-backed bat (*Pteronotus gymnonotus*) and blyth’s horseshoe bat (*Rhinolophus lepidus*) are also predominantly in the most superficial layer (ZS), with few retinal fibers distributed in the SGS layer (Cotter and Pierson Pentney, 1979), similar to the present results in *A. planirostris*.

### Final Considerations

For more than 30 years, the distribution of retinal retinal projections has been described in several species of rodents, bats, and primates via intraocular injections of tracers. This method has become a powerful tool for elucidates the organization and evolution of the visual system and also provides substantial evidence to boost new investigations. In the present study, we described a well-developed visual system in a bat species. Interestingly, no accessory optic nuclei were noted in *A. planirostris*, which raises some functional speculations on control of the compensatory eye movements. The results found in AOS in *A. planirostris* are similar to that observed in Myotis (Cotter and Pierson Pentney, 1979). The morphometrical and stereological analyses performed in this study support previous findings of functional studies, which suggest the use, to some degree at least, of vision in prey detection, spatial navigation, color perception, and roost location in bats. Notably, the dLGN in *A. planirostris* is larger than that reported in rodents. Furthermore, different from rodents, the dLGN in *A. planirostris* was shown to have two distinct layers according to the mean of the ROD measurement and qualitative analysis of retinal fibers. The previous report by Ling et al. (1997) anatomically distinguished terminals, showing R1/R2-like terminals with large varicosities and thick axons. The authors also showed string-like configurations that comprised axon collaterals studded with buttons of various sizes and simple *en passant* varicosities and terminal swellings. These anatomical findings fit the functional descriptions provided by Petrof and Sherman (2013), in which different classes of terminals induced two distinct classes of responses. Class 1 is defined as driver input, which induces paired-pulsed depression, all-or-none responses, and the absence of metabotropic components. On the other hand, class 2 of terminals is defined as modulator fibers that raise paired-pulse facilitation, graded responses and have a metabotropic component. The qualitative analysis performed in the present study connects these findings in bats. In the dLGN, PNT and SC were shown to have different classes of terminals with preferences for certain visual areas, suggesting a driver effect in some nuclei or modulatory effect in others. Taken together, these findings strongly suggest functional subdivisions in the dLGN, PNT and SC. In summary, the light intensity, color of the object, size and mobility of prey, feeding strategies of potential predators and particular aspects of the species’ ecological niche contribute to the adaptative nature of the arrangement of the cells in the visual nuclei in bats, as well as induce different wiring configurations in the retinal fibers among species. Finally, these findings in *A. planirostris* are compatible with the significance of vision in bats. These findings provide undoubted anatomical evidence that supports the use of visual cues by echolocating bats guide their behaviors.

## Author Contributions

ESN, MC and JCC participated with JSC in the conception and planning of this study. MS, PLAGM, HM and ML provided bats. MS and JGS had the primary responsibility for all aspects of bat husbandry. MB had the responsibility for all aspect of bat capturing and handling. ESN and FL supervised the tissue processing. ESN and MS supervised the photography, prepared the early drafts of the article and figures and analyzed the results. JCC, JSC and MC have read and made substantive additions and correction to final article.

## Conflict of Interest Statement

The authors declare that the research was conducted in the absence of any commercial or financial relationships that could be construed as a potential conflict of interest.

## Abbreviations

3V: 3rd ventricle
APT: Anterior pretectal nucleus
Aq: Aqueduct
CL: Centrolateral thalamic nucleus
CM: Central medial thalamic nucleus
CTb: Cholera toxin subunit B
dLGN: Dorsal lateral geniculate nucleus
DpG: Deep gray layer superior colliculus
DpWh: Deep white layer superior colliculus
fr: Fasciculus retroflexus
IAM: Interanterodorsal thalamic nucleus
InG: Intermediate gray layer superior colliculus
InWh: Intermediate white layer superior colliculus
LD: Laterodorsal thalamic nucleus
LHb: Lateral habenular nucleus
LP: Lateral posterior thalamic nucleus
LPLR: Lateral posterior thalamic nucleus, laterorostral
LPMR: Lateral posterior thalamic nucleus, mediorostral
MD: Mediodorsal thalamic nucleus
MHb: Medial habenular nucleus
MPT: Medial pretectal nucleus
OP: Optic nerve layer superior colliculus
OPT: olivary pretectal nucleus
NOT: nucleus of the optic tract
pc: Posterior commissure
Pcom: Nucleus posterior commissure
PF: Parafascicular nucleus
PNT: pretectal nuclear complex
PO: Posterior thalamic nucleus
PPT: posterior pretectal nucleus
PrC-: Precommissural nucleus
PV: Paraventricular nucleus
Re: Reuniens thalamic nucleus
Rh: Rhomboid thalamic nucleus
SC: Superior colliculus
SGS: Superficial gray superior colliculus
str: Superior thalamic radiation
vLGN: Ventral lateral geniculate nucleus
VP: Ventral posterior thalamic nucleus
ZS: Zonal layer superior colliculus.
